# Effect of optimized transcranial direct current stimulation on motor cortex activation in patients with sub-acute or chronic stroke: a study protocol for a single-blinded cross-over randomized control trial

**DOI:** 10.3389/fnins.2023.1328727

**Published:** 2023-12-21

**Authors:** TaeYeong Kim, Jhosedyn Carolaym Salazar Fajardo, Hanna Jang, Juwon Lee, Yeonkyung Kim, Gowun Kim, Donghyeon Kim

**Affiliations:** ^1^Research Institute, Neurophet Inc., Seoul, Republic of Korea; ^2^Department of Rehabilitation Medicine, Kangwon National University Hospital, Chuncheon-si, Republic of Korea

**Keywords:** brain modeling, stroke, non-invasive brain stimulation, transcranial direct current stimulation, neurorehabilitation

## Abstract

**Introduction:**

Transcranial direct current stimulation (tDCS) has shown positive but inconsistent results in stroke rehabilitation. This could be attributed to inter-individual variations in brain characteristics and stroke lesions, which limit the use of a single tDCS protocol for all post-stroke patients. Optimizing the electrode location in tDCS for each individual using magnetic resonance imaging (MRI) to generate three-dimensional computer models and calculate the electric field (E-field) induced by tDCS at a specific target point in the primary motor cortex may help reduce these inconsistencies. In stroke rehabilitation, locating the optimal position that generates a high E-field in a target area can influence motor recovery. Therefore, this study was designed to determine the effect of personalized tDCS electrode positions on hand-knob activation in post-stroke patients.

**Method:**

This is a crossover study with a sample size of 50 participants, who will be randomly assigned to one of six groups and will receive one session of either optimized-active, conventional-active, or sham tDCS, with 24 h between sessions. The tDCS parameters will be 1 mA (5 × 5 cm electrodes) for 20 min. The motor-evoked potential (MEP) will be recorded before and after each session over the target area (motor cortex hand-knob) and the MEP hotspot. The MEP amplitude at the target location will be the primary outcome.

**Discussion:**

We hypothesize that the optimized-active tDCS session would show a greater increase in MEP amplitude over the target area in patients with subacute and chronic stroke than conventional and sham tDCS sessions.

**Clinical trial registration:** https://cris.nih.go.kr, identifier KCT0007536.

## 1 Introduction

While a range of disabilities can present after stroke, the most prevalent impairment is motor deficits, affecting approximately 80% of stroke survivors (Yuan et al., 2023), and the upper extremities are generally affected with a prevalence of approximately 77% (Van Hoornweder et al., 2021). For stroke survivors, clinical interventions that enhance their functional recovery are essential to improve their quality of life (Lieshout et al., 2020; Martino Cinnera et al., 2020).

Transcranial direct stimulation (tDCS) is a non-invasive brain stimulation technique that uses a weak constant current to influence changes in cortical excitability (Laakso et al., 2019). tDCS is used to deliver an electric current through regions involved in a specific motor task, such as the primary motor cortex (M1), in post-stroke patients for motor rehabilitation (Kuo et al., 2020). Research suggests that tDCS enhances synaptic plasticity and consequently boosts motor learning (Van Hoornweder et al., 2021). Furthermore, tDCS has been shown to be effective when combined with standard physical therapy and/or other interventions used in stroke rehabilitation (Navarro-López et al., 2021; Lee and Cha, 2022). Hence, it is considered to be a promising tool for motor rehabilitation after stroke (Bornheim et al., 2022). However, despite growing evidence supporting the clinical use of tDCS in stroke rehabilitation, inconsistent results, make the implementation of tDCS in clinical settings challenging. Some studies have found that tDCS improves motor function after stroke, whereas others have found no significant effects. These inconsistencies might have been influenced by the heterogeneity of stimulation parameters and evaluation tools within studies (Santos Ferreira et al., 2019; Bornheim et al., 2022; van der Cruijsen et al., 2022; Yuan et al., 2023). Another possible reason for the inconsistent effects of tDCS in some studies on patients with stroke could be due to varying brain characteristics and stroke lesions in individuals (Evans et al., 2020). Anatomical variations in the scalp, skull, cerebrospinal thickness, cortical folding, and sulcal depth can influence the spread and intensity of the current (Laakso et al., 2015). Additionally, depending on brain tissue conductivity, lesion location, and stroke size, there may be differences in the electric current pathways within individuals who have experienced a stroke (Evans et al., 2020; van der Cruijsen et al., 2022).

The effects of tDCS are typically measured by changes in the amplitude of motor-evoked potentials (MEPs) induced by transcranial magnetic stimulation (TMS; Karatzetzou et al., 2022). According to previous research, the MEP increases when anode tDCS is applied over the M1, whereas cathode tDCS decreases MEP amplitude (Yuan et al., 2023). Additionally, a study identified that higher MEP amplitude strongly correlated with better outcomes in motor upper limb and hand recovery in patients with subacute stroke (Bembenek et al., 2020). The MEP hotspot (the scalp position at which a contralateral MEP with the maximum amplitude and lowest threshold is recorded) is believed to be the best location where anode tDCS induces changes in M1 excitability (Lee et al., 2015). Furthermore, previous research has investigated the possibility of further enhancing cortical excitability through optimized stimulation locations guided by computer modeling and electric field (E-field) magnitude. Their findings revealed that cortical excitability was more significant when the anode was positioned over the optimized area compared with the standard MEP hotspot, with an average current density significantly higher of 11.7% at the motor hand area (Lee et al., 2015).

Moreover, many clinical studies use the conventional electrode position based on the 10–20 electroencephalography (EEG) electrode guide placement to apply tDCS to improve motor skill (Rich and Gillick, 2019). A recent study conducted a brief review and found that within the published articles indexed by PubMed for the year 2022, 67.6% used this guide (Kim et al., 2023). Although the guide considers the head geometry of an individual, it does not consider many important factors that influence the E-field, such as skull thickness, white and gray matter anisotropy, cortical folding, and stroke lesion size and location (Evans et al., 2020). Recent research has proposed that montage optimization should be considered by adopting individualized models for patients with stroke (Yuan et al., 2023). The study found that anodal tDCS over M1 at 1 mA intensity for 20 min facilitates functional connectivity. It further identified the correlation between individualized E-field strength and the functional connectivity improvement in the active group, implying that E-field strength predicts functional outcomes in chronic stroke patients (Yuan et al., 2023). Consequently, the use of computational modeling and estimation of the E-field induced by tDCS can contribute to reducing the variability of tDCS effects in patients with stroke by determining the optimal electrode location to generate the best E-field magnitude over the M1.

Reducing inter-individual variability may be possible by estimating the current flow using computer modeling, such as fine element modeling (FEM; Yuan et al., 2023). FEM allows the simulation of the tDCS current-induced E-field on realistic head models while considering individual anatomical features (Saturnino et al., 2015; Yuan et al., 2023). A realistic three-dimensional (3D) head model of a person can be reconstructed using 3D T1 magnetic resonance imaging (MRI) that includes all major brain tissues (skin, skull, cerebral and cerebellar gray matter, cerebral and cerebellar white matter, cerebrospinal fluid and ventricles) and lesion area-specific conductivity assumptions (Antonenko et al., 2019). Additionally, the use of MRI can assist in predicting participants’ responsiveness to tDCS intervention by assessing their cortical thickness. This aids in categorizing potential candidates into responders and non-responders, enabling the anticipation of who might benefit from these interventions (Vaqué-Alcázar et al., 2021). A study suggests that greater structural integrity is key in predicting the effects induced by tDCS and functional connectivity evaluation showed that those who exhibited greater cortical thickness showed greater resting-State Functional MRI strength (Vaqué-Alcázar et al., 2021). Another study identified that greater gray matter volume in ipsilesional precentral/postcentral gyri and bilateral anterior cingulate cortex was associated with increased physiology predict response in post-stroke patients (Nouri and Cramer, 2011). Moreover, another study determined that the gray matter volume correlates with improvement in prefrontal tDCS treatment in patients with depression (Bulubas et al., 2019). Therefore, MRI-based analysis may prevent ineffective tDCS treatment for individuals who might not benefit from it.

Although the use of computational modeling in research and clinical practice has been recommended, its use has been scarce because it requires numerous software packages and professionals to create 3D head models and perform E-field calculations, which consumes time and resources (Yoo et al., 2022; Lee et al., 2023). Furthermore, accurate creation of a 3D head model is crucial for calculating tDCS-influenced E-field, especially as the segmentation of stroke-affected tissue directly affects the strength and orientation of the E-field in the target area (Lee et al., 2023). Recently, software capable of constructing a 3D realistic head model and accurately calculating the tDCS-induced E-field in patients with stroke in a relatively short period of time has been developed. This software allows the user to determine the optimal electrode position for each individual according to brain structure, stroke lesion, electrical conductivity, and electrode properties (Yoon et al., 2023).

Therefore,, the aim of the present crossover randomized control trial is to determine the immediate effect of personalized tDCS electrode positioning on the activation of the cortical region responsible for finger movement in patients with sub-acute or chronic stroke using MEP. Our hypothesis is that compared to conventional and sham stimulation, personalized electrode positioning will show changes in MEP amplitude over the cortical region responsible for finger movement in this population. Furthermore, we hypothesize that the participants would tolerate the tDCS sessions without experiencing any significant adverse events. We aim to reduce the inconsistencies in tDCS intervention in patients with stroke and to improve motor function in these patients, especially in the upper extremity.

## 2 Methods and analysis

### 2.1 Trial design

**Figure 1:**
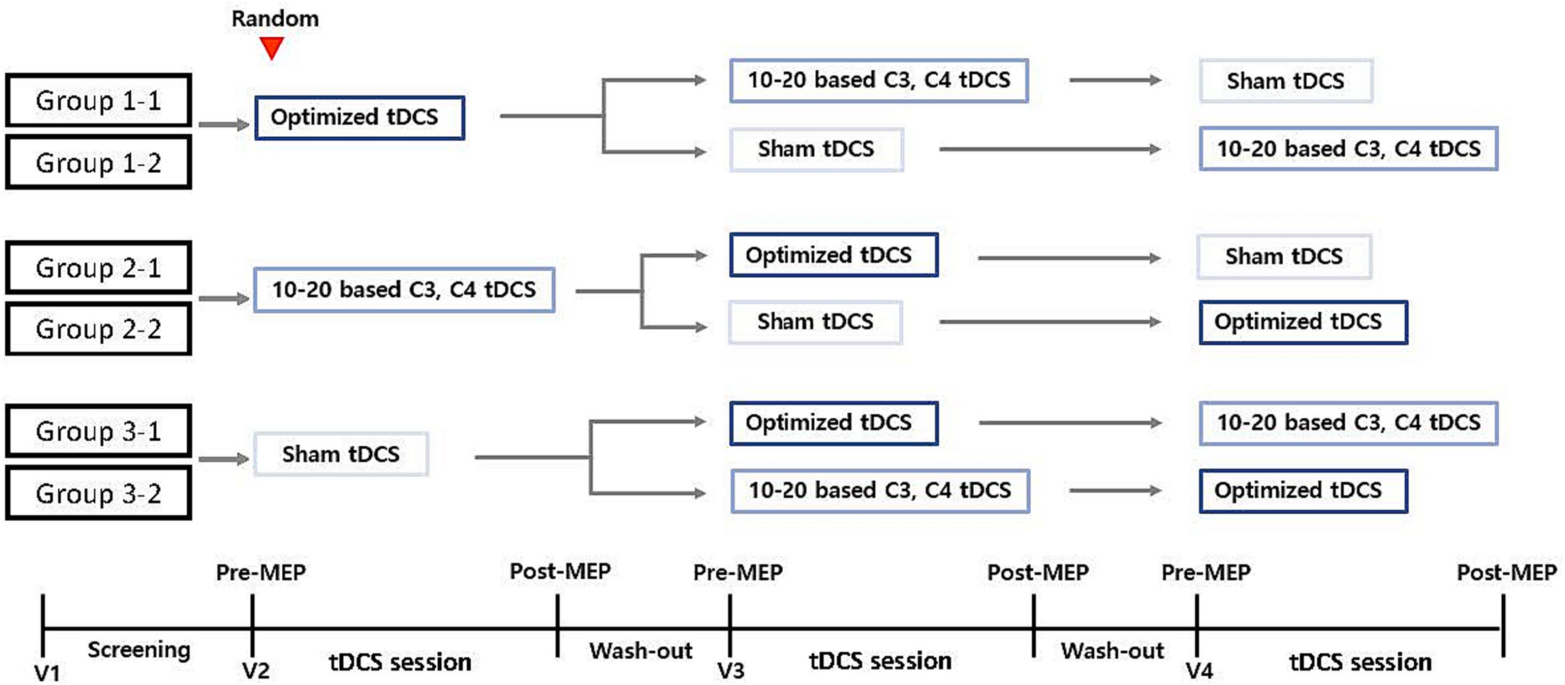
Study design. Group participants randomization into six groups for the purpose of the three different types of tDCS intervention: 1-1, 2-1, 2-2, 3-1, 3-2. MEP, motor-evoked potential; tDCS, transcranial direct current stimulation; V, visit.

The present study protocol is a prospective single-center crossover randomized control trial designed to determine the effect of personalized tDCS electrode positioning on the activation of the cortical region responsible for finger movement in patients with stroke. The crossover design will be divided into six groups (four groups of eight participants each and two groups of nine participants each). All participants will randomly receive one session of conventional-active, optimized-active, or sham tDCS. After a 24-h washout period, the groups will cross to receive a different tDCS intervention until each group receives all three alternatives. Figure 1 provides a detailed explanation of this procedure and Table 1 displays the activities to be completed at each visit during the trial.

**Table 1:** Schedule of enrollment, interventions, and assessments.

Schedule	Screening	Baseline	Wash-out (>24 h)	Assessment	Wash-out (>24 h)	Assessment
visit (V)	V1	V2		V3		V4
Written consent	V					
Demographic information	V					
Medical and surgical history	V					
Eligibility screen	V	V				
Vital signs screen	V					
Physical examination	V					
MRI examination	V					
Randomization		V				
tDCS application		V		V		V
MEP examination	V	V		V		V
Adverse events examination		V		V		V
Allowed medication and therapies screening		V		V		V

MEP, Motor-evoked potential; MRI, Magnetic resonance imaging; tDCS, transcranial direct current stimulation.

### 2.2 Recruitment and study design

The study will be performed at the Kangwon National University Hospital in Chuncheon, Republic of Korea. The hospital will screen and recruit volunteer patients who meet all the inclusion and none of the exclusion criteria.

### 2.3 Participants

#### 2.3.1 Ethics approval

The study protocol was registered under the number KCT0007536 and will be conducted according to the 1964 Declaration of Helsinki. This study was approved by the Ethics Review Board of the Kangwon National University Hospital (approval number: KNUH-2022-05-008).

A signed informed consent from the participants will be obtained after providing them with a complete explanation of the study’s objectives, benefits, and any potential discomfort that they might experience during the intervention. The participants can terminate the trial at any given moment without penalty.

#### 2.3.2 Inclusion and exclusion criteria

The inclusion criteria for this study are (1) men and women > 19 years old; (2) patients with sub-acute or chronic stroke with 4 weeks or more after onset; and (3) individuals capable of measuring MEP amplitude and latency in the M1 hand-knob region over the lesioned side, as identified on T1 MR images.

The exclusion criteria are as follows: (1) individuals with structural lesions of the brain other than cerebral infarction or cerebral hemorrhage (e.g., brain tumor, traumatic injury, etc.), or a history of brain surgery resulting in significant structural changes in the brain; (2) individuals that meet the contraindications of using tDCS, such as: deformities, inflammatory reactions, or other dermatologic problems at the electrode attachment site that could interfere with tDCS electrode attachment, presence of metallic materials on the skull area where the tDCS will be applied, and having an artificial pacemaker, artificial valve, or ear implant (e.g., cochlear implant); (3) individuals with a T1 MR with poor quality image due to image shaking, shading, or artifact noise that cannot be read or processed by the software; (4) individuals with traumatic brain injury, spinal cord injury, degenerative brain disease such as Parkinson’s disease, upper extremity nerve injury, and other conditions that may affect upper limb function; (5) individuals whose stroke cannot be identified on the T1 MRI; (6) individuals with a serious neurological disorder that is accompanied by a major psychiatric disorder, such as schizophrenia or bipolar disorder; and (7) individuals with medical contraindication for MRI.

#### 2.3.3 Sample size

G*Power 3.1.9.7 (Franz Faul, Kiel, Germany) was used to determine the sample size. It was calculated based on the effect size determined by Lee et al. in 2015 for the pre-and post-tDCS intervention and MEP amplitude results (Lee et al., 2015). It was calculated using analysis of variance (ANOVA): repeated measures, within-between interaction, with a Type I error rate of 5% and a power of 95%, and with an effect size f of 0.2193764. Consequently, a sample size of 45 participants was required to determine the effectiveness of the intervention. A dropout rate of 10% was considered. Accordingly, 50 participants will be recruited for this study.

#### 2.3.4 Randomization

Participants who meet the inclusion and none of the exclusion criteria will be randomly assigned to one of six groups. A 1:1 random ratio is used to locate the participants in each group (Figure 1). Due to the required sample size for this study, some groups will have an unequal number of participants. An independent statistician will issue randomization numbers.

### 2.4 Intervention

#### 2.4.1 Transcranial direct current stimulation

A portable battery-operated Neurophet Innk tDCS device (Neurophet, Seoul, Republic of Korea) will be utilized during the intervention. The device can be programmed to supply direct current for a specific period and intensity (1–2 mA). It is also pre-programmed to follow a ramping protocol that involves increasing the current in 30 s to reduce discomfort at the start of the intervention and decreasing the current in 30 s to conclude it. Additionally, the device detects unsafe changes in impedance values on the skin during the intervention, delivering a warning sound and terminating the stimuli when detecting an impedance of more than 13 kΩ. This function reduces adverse events that can occur during tDCS.

For this study, the device will be programmed to deliver a current of 1 mA for 20 min using 5 × 5 sponge-coated electrodes in active sessions. In the sham session, to provide the initial and final sensations of tDCS, the ramping protocol will be followed at the beginning and end of the intervention: the current will be ramped up to 1 mA in 30 s and then decreased to 0 mA in 30 s, after 18 min the ramping protocol will be repeated.

During the study, the participants will continue the clinical rehabilitation programs that were initiated before recruitment. Regarding medications, at the discretion of the investigator, participants taking Na + or Ca++ channel blockers (e.g., carbamazepine or verapamil), N-methyl-D-aspartate receptor antagonists (e.g., memantine) might be allowed to participate in the present study as long as they are under a stable dose during the totality of the study.

#### 2.4.2 Brain modeling

To determine the electrode location for stimulating the optimal tDCS-induced E-field, each participant will undergo a T1-weighted MRI session at baseline. Subsequently, a software tool called NEUROPHET tES LAB (Neurophet, Seoul, Republic of Korea) will generate a 3D brain model using the acquired images and then proceed to analyze the optimized electrode location and tDCS-induced E-field.

**Figure 2:**
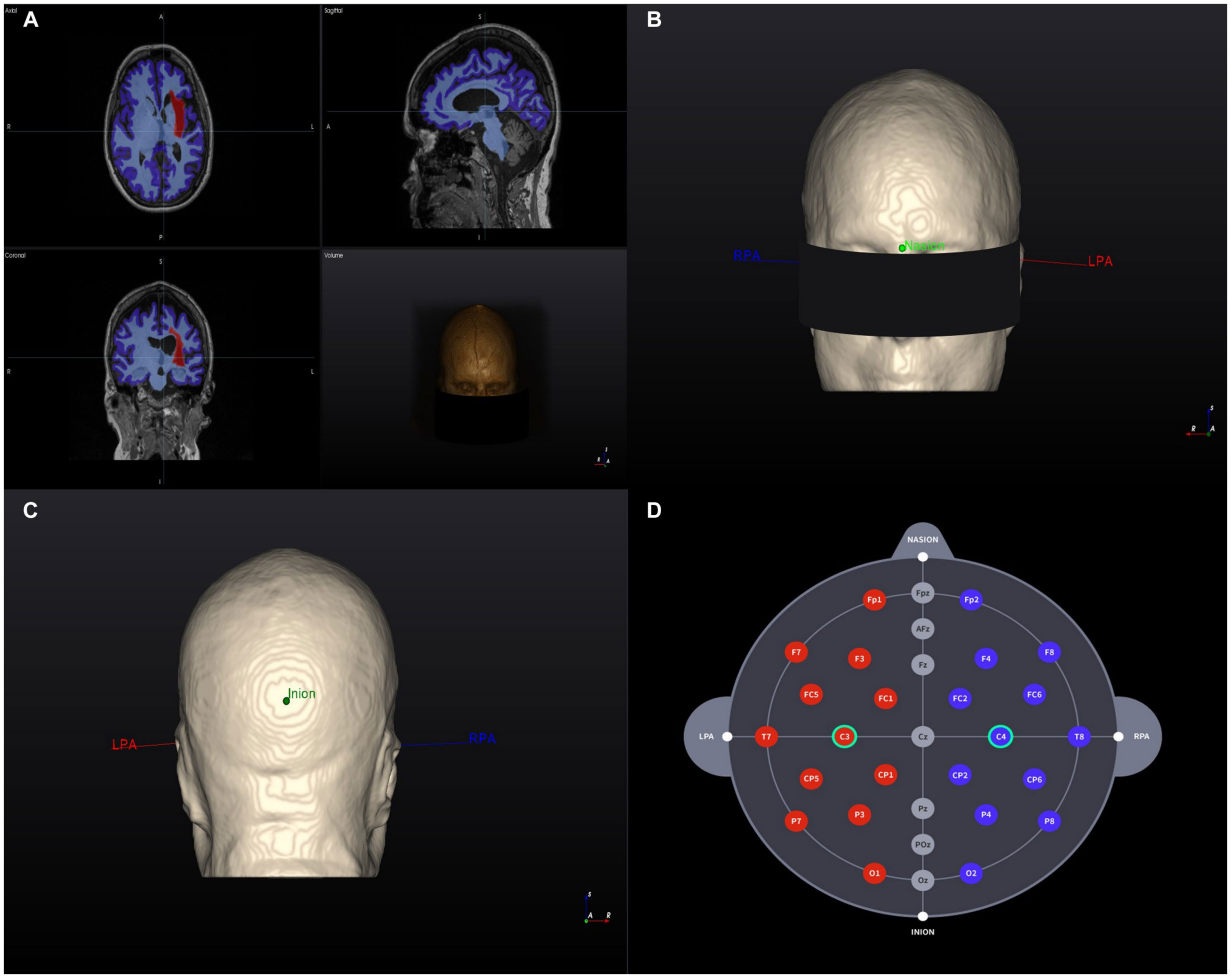
3D Brain model creation and landmarks. **(A)** T1-weighted MR image of a head model (example) and segmentation results based on tissue electrical conductivity. The red area on the left represents the stroke lesion. **(B)** Anterior view of the 3D brain model. RPA and LPA indicate the landmark locations. **(C)** Posterior view of the 3D brain model. RPA and LPA indicate the landmark locations. **(D)** Individualized selection of tDCS electrode positions (C3–C4). RPA, right prearticular; LPA, Left prearticular. Example data were randomly selected from the Anatomical Tracings of the Lesions After Stroke (ATLAS) public dataset (Liew et al., 2022).

The software analyzes and segments T1-weighted MRI data and reconstructs them into a 3D model with the following structures and pre-programmed electrical conductivity: skin (0.465 S/m), skull (0.010 S/m), cerebral and cerebellar gray matter (0. 276 S/m), cerebral and cerebellar white matter (0.126 S/m), cerebrospinal fluid and ventricles (1.65 S/m), and the stroke lesion area (0.809 S/m; McCann et al., 2019; Yoo et al., 2022). Then, an investigator will assign the landmarks (nasion, inion, and preauricular points) on the model and the software will automatically allocate the 10–20 EEG-based coordinates on the 3D model (Figure 2). The subsequent steps are performed in accordance with the tDCS sessions.

#### 2.4.3 tDCS sessions

**Figure 3:**
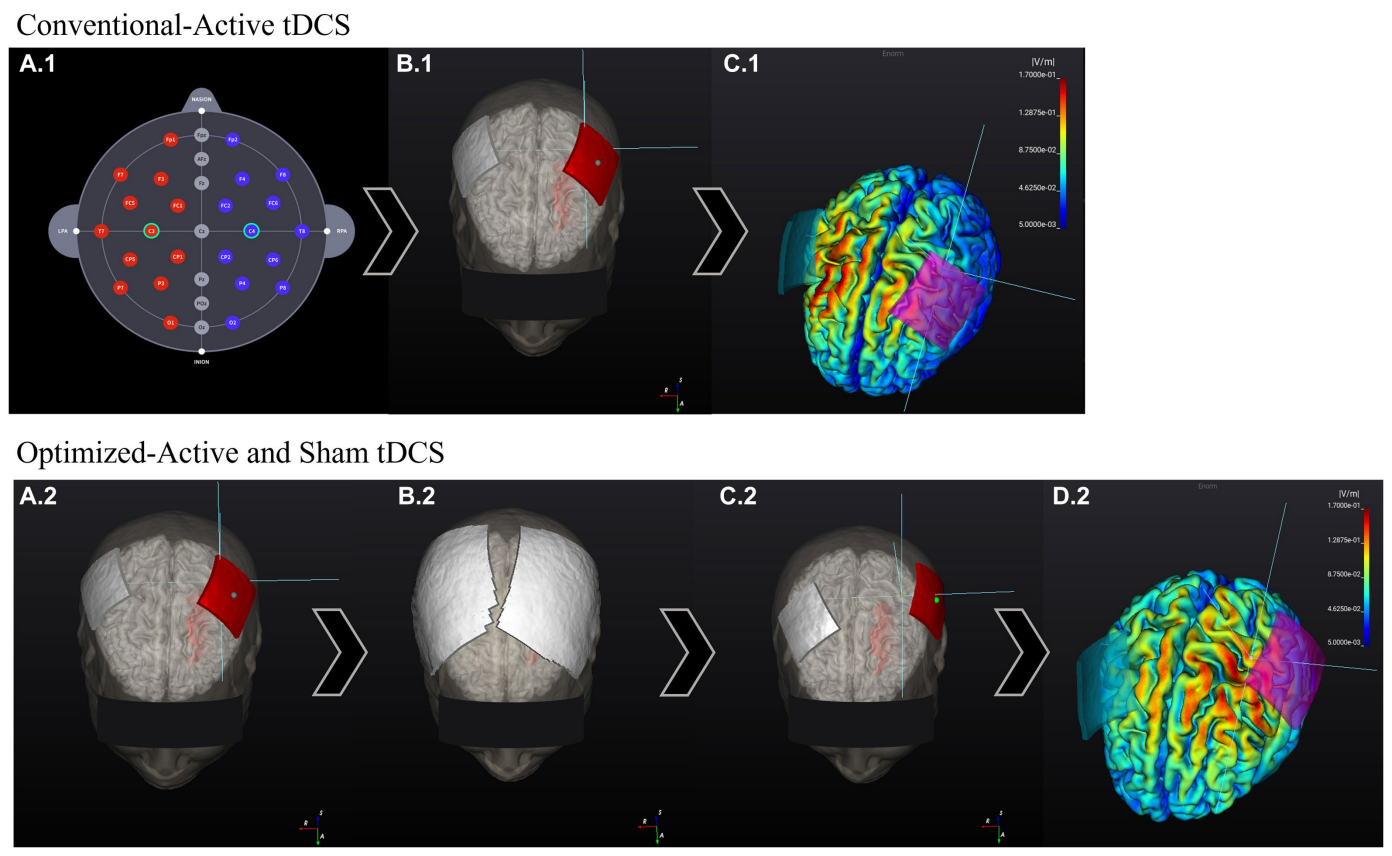
Comparison between conventional 10–20 EEG system-based electrode location E-field and optimized-tDCS electrode location E-field. The stroke lesion is located on the left side of the brain (head model). Conventional-active session: **(A.1)** Electrodes are located following the 10–20 EEG system electrode location. **(B.1)** Program positions the electrodes on the assigned areas: C3 (anode, red) and C4 (cathode, gray). **(C.1)** The program calculated the E-field generated according to the input stimulation parameters. The E-field magnitude in the target zone (M1 hand-knob) was: 0.06 V/m. Optimized-active and sham tDCS sessions: **(A.2)** The stimulation target area is input into the program, and the electrodes are initially located over the 3D-Brain model in the same way as previously explained. **(B.2)** Next, according to the input stimulation parameters, the program calculates the best electrode position that generates the highest E-field over the target area. **(C.2)** After the calculations, the program shows the new personalized electrode positions. **(D.2)** E-field generated according to the input stimulation parameters and new electrode position. E-field magnitude at the target zone (M1 hand-knob): 0.11 V/m.

**Figure 4:**
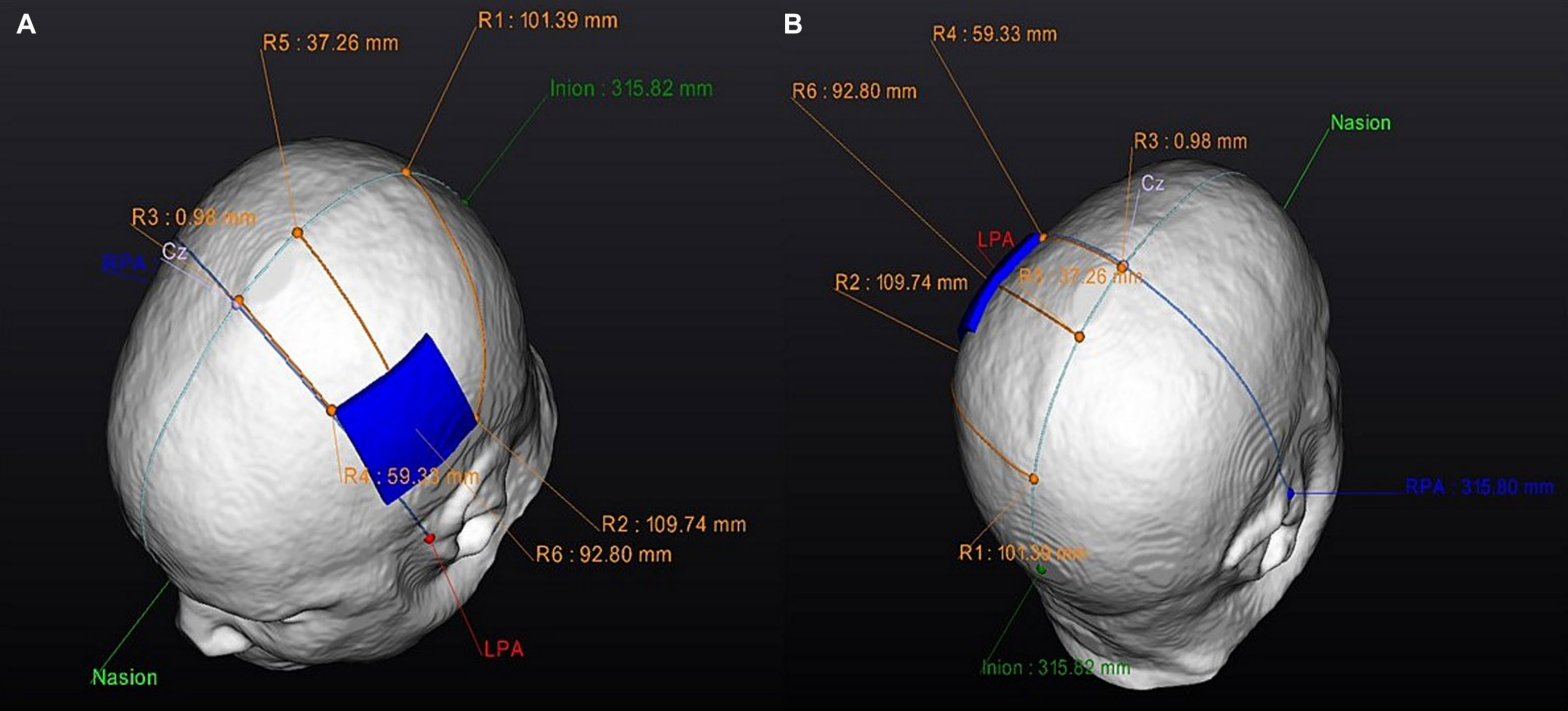
Personalized anode optimized electrode position: Electrode guides. Superior view of the optimized anode-location guide: **(A)** Left side. **(B)** Right side. RPA, right pre-articular; LPA, left pre-articular.

For the optimized-active tDCS session, the investigator will use NEUROPHET tES LAB software to determine the optimal electrode location that best stimulates the target area. For this study, the tDCS target location is the structural ipsilesional M1 hand-knob area, which will be identified on the gray matter of the 3D model by a single investigator (medical doctor experienced in stroke rehabilitation) for all the participants. Localized in a specific segment of the precentral gyrus, the hand-knob will be identified on the axial plane as a visible omega or epsilon-shaped bulge area (Yousry et al., 1997). Following the 10–20 EEG system, the anode will be placed over the ipsilesional side of the stroke lesion (C4 or C3 area), and the cathode will be positioned over the contralesional side of the 3D brain model. For example: If the participant’s stroke lesion is located over the left hemisphere the anode will be located over C3 while the cathode will be located over C4. The 3D tDCS electrodes will measure 5×5 cm and have an intensity of 1 mA. Based on the input parameters, individual brain characteristics, and tissue conductivity, the software will analyze the best potential electrode location that generates the maximum tDCS-induced E-field in the selected M1 hand-knob area, and to determine an optimized electrode location per person (Figure 3). Following the electrode guidelines provided by the software, the investigator will find the optimized locations of the anode and cathode electrodes and apply them to the participants during the session (Figure 4).

For the conventional-active tDCS session, an electrode optimization analysis will not be performed. The electrode locations are based on classic electrode positions using the 10–20 EEG system coordinates. Based on the 3D brain model of each participant, the investigator will apply the anode over the ipsilesional side of the stroke lesion (C3 or C4 area) and the cathode over the contralesional side (Figure 3). Subsequently, the software will indicate electrode guides to locate these positions on the participant’s head and proceed to conduct the session.

During the sham session, the optimized locations will be used to locate the electrodes; however, the tDCS device will follow the sham protocol described previously.

### 2.5 Blinding

Participants will be blinded to their assigned groups and the order in which they will receive the tDCS interventions. The investigator in charge of performing the MEP measurements will be unaware of the participant allocation during the measurements.

As the parameters of this study are about individualized tDCS electrode location, and due to the nature of the tDCS device programming application, one investigator will remain unblinded to the group’s allocation. This investigator will be called the “Investigational device manager,” who will be responsible for applying the tDCS to the participants during the study but will not be involved in other parts of the study.

### 2.6 Outcome measurement

#### 2.6.1 Motor-evoked potential

In this study, MEP will be induced by the application of TMS over the M1 after positioning the center of the coil at 45° lateral to the sagittal midline (Muellbacher et al., 2000). An optimal TMS position is chosen to record the maximum potential amplitude of the abductor pollicis brevis (APB), identified as the hotspot area, which will be called conventional hotspot area for this trial (Muellbacher et al., 2000). The stimulation intensity of the M1 is determined by motor threshold, which is the intensity at which the first potential or muscle contraction occurs, starting at 40% of the maximum intensity of the stimulator and is increased by 5%.

The MEP amplitude and latency will be assessed. Latency is defined as the period from the start of a single-pulse TMS to the emergence of a wave peak in milliseconds, whereas amplitude is the distance from the baseline to the wave peak in microvolts (Lee et al., 2015).

The MEP is assessed immediately before and after each tDCS session. Hence, each group will be assessed six times over the course of the trial by investigators who are blinded to the group allocation.

#### 2.6.2 Primary outcome

- The difference between groups in MEP amplitude recorded before and after each tDCS session (optimized-active, conventional-active and sham tDCS) over the M1 hand-knob area selected by the investigator on the 3D brain model of the tES LAB.

#### 2.6.3 Secondary outcomes

- The difference between groups in the MEP amplitude over the conventional hotspot recorded before and after each tDCS session (optimized-active, conventional-active and sham tDCS).
- The MEP latency changes between groups recorded before and after each tDCS session (optimized-active, conventional-active and sham tDCS) over the conventional hotspot area and the M1 hand-knob area selected by the investigator on the 3D brain model of the tES LAB.
- Correlation between MEP changes before and after each tDCS session (optimized-active, conventional-active and sham tDCS) over the conventional hotspot area and the M1 hand-knob area selected by the investigator on the 3D brain model of the tES LAB, and the E-field generated at the structural M1 hand-knob.

### 2.7 Statistical analyses

The results of the outcome assessment analysis will be presented as mean, standard deviation (SD), standard error (SE), and confidence interval, with the statistical significance threshold set at *p* < 0.05. The Shapiro–Wilk test will be employed to assess the normality of the data. If normality is met, a repeated measures ANOVA test will be used for within-and in-between groups analysis, and Tukey’s test will be used for *post hoc* analyses. If normality is not met, the Friedman test will be used with the Wilcoxon rank-sum test to compare independent variables. Additionally, the correlation between the pre-post MEP conventional hotspot and the M1 hand-knob area selected by the investigator on the 3D brain model of the tES LAB with the E-field generated at the structural M1 hand-knob area will be analyzed.

### 2.8 Safety, protocol, and data monitoring

During and 30 min after stimulation, the researchers will verify whether any adverse events related to tDCS, such as fatigue, dizziness, and redness, will present. If any adverse events occur, they will be assessed by a physician, classified according to severity criteria (mild, moderate, and severe), and report to the Institutional Review Board (IRB).

### 2.9 Trial status

Participant recruitment began in September 2022, and the study is still in the recruitment phase of completing the required sample size.

## 3 Discussion

The present crossover randomized control trial aims to determine the effect of personalized tDCS electrode positioning on the activation of the cortical region responsible for finger movement in patients with sub-acute or chronic stroke. The study design utilizes MEP data and 3D brain models derived from individual MRI scans to determine optimized tDCS electrode locations and compares them with conventional 10–20 EEG system electrode locations.

In general, anodal tDCS with low-intensity stimulation increases corticospinal excitability, whereas cathode stimulation may decrease it (Reed and Cohen Kadosh, 2018). However, tDCS has considerable inter-patient variability and a partially non-linear dose-dependent effect, as factors such as stimulation intensity and duration can diminish or reverse these effects (Laakso et al., 2015; Hassanzahraee et al., 2020). In patients with stroke, anode tDCS is frequently applied over the ipsilesional hemisphere, according to the interhemispheric competition model (Van Hoornweder et al., 2021). In this model, the underactive affected hemisphere experiences an inhibitory influence from the overactive unaffected hemisphere after a stroke (Di Pino et al., 2014). Consequently, the ipsilesional hemisphere is not only disabled due to stroke-induced tissue damage but also due to extensive interhemispheric inhibition (Van Hoornweder et al., 2021). Following this model, anodal tDCS increases cortical excitability by applying anode over the ipsilesional region and decreases contralesional cortical excitability through cathodal stimulation or through a bihemispheric electrode montage.

A study focused on the effect of tDCS on upper extremity function in patients with acute, sub-acute, or chronic stroke indicated that tDCS significantly improves upper limb function in patients with chronic stroke. In particular, bihemispheric tDCS seemed to have a fairly large effect on motor recovery of the upper extremity (Van Hoornweder et al., 2021). Although tDCS has been shown to be a promising tool to improve motor recovery after stroke, it is not yet widely used in clinical practice because of inconsistent effects reported in various meta-analyses (Van Hoornweder et al., 2021; Bornheim et al., 2022; van der Cruijsen et al., 2022). Currently, conventional tDCS results show variable E-fields within the motor cortex in patients with chronic stroke (Laakso et al., 2015).

Patient dependent volume conduction effects determine passage of the current throughout the head and are affected by structural brain changes, such as stroke lesions (Piastra et al., 2021). Computer modeling simulations with volume conduction models that include lesions might facilitate the effective application of tDCS in stroke rehabilitation, thereby enhancing the impact of tDCS and reducing the inconsistencies found in the research to date.

A recent study investigated the E-field strengths generated by conventional and optimized tDCS configurations in patients with stroke and healthy age-matched participants using computer head simulations. The results showed that, when electrode locations were optimized, the electrode configurations resulted in a greater variety of electrode settings in patients with stroke than in healthy participants, likely because of the presence of stroke lesions. These findings suggest that the inter-individual variability in E-field strengths may have contributed to the lack of beneficial effects of tDCS found in clinical trials and that considering individual brain structure and functional motor targets is vital for utilizing tDCS in patients with stroke for rehabilitation (van der Cruijsen et al., 2022).

The study design aims to assess the use of individualized, optimized tDCS electrode locations (generated by a computer brain modeling simulation created using 3D T1 MRI) and compare them with conventional and sham tDCS in real clinical settings. Additionally, we expect to include a relatively large sample size (for tDCS studies) of 50 post-stroke participants. It is expected that this research will contribute to reducing the variability of results found in previous research and promote the use of individualized tDCS electrode locations in future clinical research.

However, this study presents some limitations. First, only the immediate effects of tDCS will be evaluated as the participants will be assessed immediately after the application. Therefore, the short-and long-term effects of tDCS should be investigated in future studies. Second, the study will include and analyze data combined from patients with sub-acute or chronic stroke, which should be interpreted independently in future research to determine the effect of optimized tDCS electrode locations according to each stroke phase. Furthermore, inclusion of patients with acute stroke is necessary. Third, the study sample includes participants within a wide age range (young and older adults), which may influence the results.

As expressed before, we hypothesize that the optimized active tDCS session will show a greater increase in MEP over the cortical region responsible for finger movement in patients with sub-acute or chronic stroke than conventional and sham tDCS sessions. Additionally, we hypothesized that participants would tolerate the tDCS sessions without any significant adverse events.

## Ethics statement

The trial protocol was reviewed and approved by Ethics Review Board of the Kangwon National University Hospital (approval number: KNUH-2022-05-008). The participants will provide their full informed consent to participate in this study.

## Author contributions

TK: Conceptualization, Methodology, Writing – original draft, Funding acquisition. JCSF: Conceptualization, Methodology, Writing – original draft. HJ: Conceptualization, Methodology, Writing – original draft. JL: Methodology, Writing – review & editing. YK: Methodology, Writing – review & editing. GK: Methodology, Writing – review & editing. DK: Conceptualization, Funding acquisition, Methodology, Writing – original draft.
